# SF3B1 is a stress-sensitive splicing factor that regulates both HSF1 concentration and activity

**DOI:** 10.1371/journal.pone.0176382

**Published:** 2017-04-26

**Authors:** Karen S. Kim Guisbert, Eric Guisbert

**Affiliations:** Department of Biological Sciences, Florida Institute of Technology, Melbourne, FL, United States of America; Washington State University, UNITED STATES

## Abstract

The heat shock response (HSR) is a well-conserved, cytoprotective stress response that activates the HSF1 transcription factor. During severe stress, cells inhibit mRNA splicing which also serves a cytoprotective function via inhibition of gene expression. Despite their functional interconnectedness, there have not been any previous reports of crosstalk between these two pathways. In a genetic screen, we identified SF3B1, a core component of the U2 snRNP subunit of the spliceosome, as a regulator of the heat shock response in *Caenorhabditis elegans*. Here, we show that this regulatory connection is conserved in cultured human cells and that there are at least two distinct pathways by which SF3B1 can regulate the HSR. First, inhibition of SF3B1 with moderate levels of Pladienolide B, a previously established small molecule inhibitor of SF3B1, affects the transcriptional activation of HSF1, the transcription factor that mediates the HSR. However, both higher levels of Pladienolide B and SF3B1 siRNA knockdown also change the concentration of HSF1, a form of HSR regulation that has not been previously documented during normal physiology but is observed in some forms of cancer. Intriguingly, mutations in SF3B1 have also been associated with several distinct types of cancer. Finally, we show that regulation of alternative splicing by SF3B1 is sensitive to temperature, providing a new mechanism by which temperature stress can remodel the transcriptome.

## Introduction

The heat shock response (HSR) was first identified more than fifty years ago as an increase in the puffing of *Drosophila* salivary chromosomes in response to elevated temperature [1]. Since then, the HSR has been established as a cellular stress response that senses protein folding and leads to activation of the HSF1 transcription factor and the upregulation of a set of cytoprotective heat shock genes (reviewed in [2]). Interest in the HSR has increased in recent years due to its emerging roles in the regulation of lifespan, protection against neurodegenerative diseases, and its important connection with several different types of cancer (reviewed in [3,4]).

HSF1 is regulated through several distinct mechanisms. Primary stress sensing occurs through a negative feedback loop with the HSP70 and HSP90 chaperone machines that can directly bind to HSF1 and repress its activity [5,6]. HSR regulation involves repression of HSF1 by HSP90 and HSP70 when misfolded proteins are low and chaperones are in excess. When misfolded proteins accumulate, chaperones are titrated away from HSF1, initiating the HSR. The HSF1 activation pathway includes several other steps including extensive post-translational modification, translocation to the nucleus, trimerization, and derepression of the HSF1 transcriptional activation domain.

Increased temperature leads to several other cellular effects not mediated by HSF1. For example, inhibition of mRNA splicing occurs upon exposure to severe, but not mild, heat stress in *Drosophila* and cultured human cells [7,8]. One mechanism proposed to mediate heat-induced inhibition of mRNA splicing is dephosphorylation of SRp38, which associates with the U1 subunit of the spliceosome and disrupts its ability to recognize pre-mRNA [9,10]. Severe heat shock has also been shown to inhibit protein translation through phosphorylation of eIF-2α[11] and relocalization of mRNAs into cytoplasmic stress granules [12]. These distinct mechanisms work together to reduce new protein synthesis, which in concert with induction of heat shock proteins, function to promote protein folding. In each case, HSR-induced genes are able to escape the bulk inhibition of gene expression. Despite their mutual importance, there have not been any previous reports of crosstalk between the cellular pathways that inhibit gene expression and the protective HSR.

Using a genome-wide screen in *C*. *elegans*, we have identified a network of HSR regulators [13]. Intriguingly, one of the positive HSR regulators, SF3B1, is an mRNA splicing factor, introducing the possibility that regulation of the HSR is linked to inhibition of mRNA splicing during stress. SF3B1, first described as SAP155, is the largest member of the SF3B complex, a core component of the U2 snRNP [14–16]. SF3B1 is in the privileged position of directly contacting the substrate mRNA and phosphorylation of SF3B1 occurs concomitantly with splicing catalysis [17]. The C terminus of SF3B1 has an extended domain of 22 HEAT repeats that form a ladder-like structure important for interacting with other members of the SF3B complex and additional splicing factors [18–20]. Although SF3B1 is a core member of the splicing machinery, inhibition of SF3B1 does not cause a bulk defect in splicing *in vivo*, rather SF3B1 has a critical role in alternative splicing and 3’ splice site selection [21–23].

Importantly, SF3B1 is mutated in several types of cancer, including chronic lymphocytic leukemia, uveal melanoma, breast, ovarian, and pancreatic cancers (reviewed in [24]). Analysis of genes mutated across 21 cancer types revealed that SF3B1 is among a group of genes that are most strongly associated with cancer [25]. In addition to the high incidences of SF3B1 mutations, several small molecules with anticancer activity have been found to specifically target SF3B1 [24]. For example, Pladienolide B is a compound from *Streptomyces platensis* that targets SF3B1 and is able to selectively kill cancer cells [26].

The intriguing link between the mRNA splicing machinery and the heat shock response and the important roles of both SF3B1 and HSF1 in cancer prompted us to further analyze the connections between SF3B1 and the HSR. Here, we demonstrate that this regulation is conserved in human cells, delineate two pathways by which SF3B1 regulates HSF1, and discover that regulation of alternative splicing by SF3B1 is sensitive to stress.

## Materials and methods

### Cell culture

HeLa cells were grown in DMEM or MEM Alpha with 10% FBS at 37° with 5% CO_2._ Cells were maintained between 5–95% confluency. Experiments were performed on cells passaged no more than 17 times. Cells were grown in a 25cm^2^ flask, 100mm dish or 6-well dish. The cell line was a gift from the laboratory of Richard Morimoto. Pladienolide B was purchased from Santa Cruz Biotechnology and was dosed at concentrations ranging from 1nM to 100nM.

### RNAi knockdowns

Cells were grown to confluency in 100mm dishes. Cells were trypsinized, washed and incubated in 6-well dishes. Cells were transfected using RNAiMAX from Invitrogen and then incubated for 2 days for all siRNA experiments. For heat shocked cells, the edges of the 6-well dishes were wrapped at least 3 times with parafilm, taking care not to touch the wells themselves, and submerged in a pre-equilibrated 42° water bath for 1 hour. After the shock, cells were immediately harvested for RNA extraction or recovered for four hours at 37° for western blot analysis. All experiments were performed with biological triplicates unless otherwise indicated.

### RNA extraction and analysis

RNA was extracted from cells grown in 6-well dishes by direct lysis into Trizol Reagent from Life Technologies. After room temperature incubation for 5 minutes, the cells were transferred to a microcentrifuge tube with 200μl of chloroform. The mixture was incubated for 5 minutes at room temperature then centrifuged at 4°C for 15 minutes. The supernatant was moved to a fresh tube containing 500μl of isopropanol for RNA precipitation. The alcohol mixture was chilled and then centrifuged at 4°C for 20 minutes. The RNA pellet was washed with 70% ethanol and air dried. The RNA pellet was resuspended in water and quantified using a NanoDrop Lite.

The RNA was normalized using total RNA concentration and treated with DNaseI using the DNA-free kit from Ambion. After DNaseI inactivation, RNA was reverse transcribed using the iScript kit from Bio-Rad. cDNA was subjected to qRT-PCR analysis using the iQ supermix from Bio-Rad and amplified using the MyiQ real-time cycler from Bio-Rad or the CFX Connect cycler from Bio-Rad. All reactions were performed with an annealing temperature of 58°C. All qPCR experiments were performed with at least technical duplicates and biological triplicates. p-values were calculated using the student’s t-test. All primers are listed in Table 1.

**Table 1:** List of oligonucleotide primers.

*ACTB* exon 4, forward	AGCGGGAAATCGTGCGTGAC
*ACTB* exon 4, reverse	GGAAGGAAGGCTGGAAGAGTGC
*ACTB* exon 5, forward	CCTGGCACCCAGCACAAT
*ACTB*, exon 6, reverse	GGGCCGGACTCGTCATAC
*DNAJB1* exon 2, forward	CCTTTTTTGGGCAGCGGAAC
*DNAJB1* exon 2, reverse	ATCTTGCTTCTTTCGGGCGG
*HSF1* exon 6, forward	TCTCACTGGTGCAGTCAAAC
*HSF1* exon 7, reverse	GGCTATACTTGGGCATGGAAT
*HSP70A6* forward	ACCACCTACTCGGACAACC
*HSP70A6* reverse	CACGCTCAGGATGCCATTAG
*RBM5* exon 7, forward	TTGGTGATTCAAGGAAAGCA
*RBM5* exon 5–7, forward	CTGATGAAGAGGAAAACAGAAAAAG
*RBM5* exon 7, reverse	CAAAGCCAATCTTCAAACTTAGG
*SF3B1* exon 19, forward	ACGGCTTTGGCACAGTGGTTAATG
*SF3B1* exon 19, reverse	AAATCAAGTCAGCTGCCTGTTGCC

### Westerns

Samples were harvested from cells grown in 6-well dishes using a cell scraper into DPBS. Cells were then transferred to a microcentrifuge tube and collected with a brief spin. DPBS was removed and 100μl of 2X sample buffer was added directly to the cell pellet. The mixture was immediately placed in a boiling water bath for at least 10 minutes with intermittent vortex mixing.

SDS-PAGE gels with 10 wells were poured with a 9% resolve and a 3% stack. Dual Precision Plus standards from Bio-Rad were run on every gel. After separation, the proteins were transferred to nitrocellulose using the wet transfer method and blocked with 5% nonfat milk. Primary antibody incubations were performed for at least 1 hour in TBST with 5% nonfat milk. Mouse monoclonal antibodies (Sigma, F3777, antibody ID AB_476977) were used to detect ACTIN at 1:5000 dilution. Rabbit polyclonal antibodies were used to detect HSF1 at 1:10,000 dilution [27]. Rabbit polyclonal antibodies (Sigma, SAB2102122, antibody ID AB_10605203) were used to detect SF3B1 at 1:1000 dilution. Mouse monoclonal antibodies (Santa Cruz Biotech, SC-66048, antibody ID AB_832518) were used to detect HSP70 at 1:500 dilution. Secondary antibody incubations were performed for no longer than 30 minutes in TBST with 5% nonfat milk at 1:10,000 dilution. At least 30 minutes of washes in TBST were performed after each antibody with frequent buffer changes using at least 400ml of TBST per blot. SuperSignal West Femto chemiluminescent kit from Life Technologies was used to detect the secondary antibody. All westerns included at least two independent biological replicates.

### Luciferase

7000 cells per well were grown in standard 96-well opaque plate. Cells were transfected with the luciferase reporter under the Gal4 promoter, a NanoLuc (Promega) normalization control with a TK (thymidine kinase) promoter and either the Gal4-HSF1 chimera or the positive control Gal4-VP16 [28]. Cells were grown for 4 hours, then treated with either 2nM or 100nM Pladienolide B for 16 hours. Cells were heat shocked for 1 hour at 42°, allowed to recover at 37° for 7 hours, then quantitated using the Nano-Glo Dual-Luciferase Reporter Assay (Promega). The 7 hour incubation after heat shock was used to maximize luciferase activity as it was previously shown that luciferase folding and activity is inhibited immediately after heat shock [29]. Signals were read on a SpectraMax i3 from Molecular Devices and integrated over 1000 msec. Dual-luciferase using NanoLuc and a VP16-Gal4 control were used to normalize the data to account for transfection efficiency, cell density, and any non-specific effects of PB on luciferase. All experiments were performed with biological triplicates.

## Results

### SF3B1 is a positive regulator of the heat shock response in human cells

**Fig 1:**
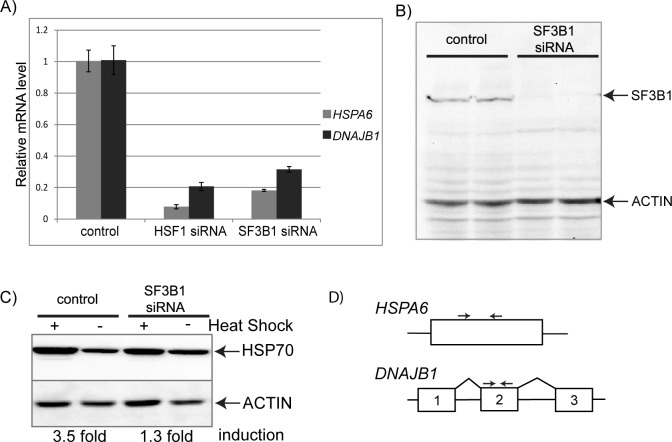
Depletion of SF3B1 inhibits the HSR. (A) qRT-PCR analysis of mRNA levels of two heat shock genes, *HSPA6* and *DNAJB1*, in cells that have been treated with either *HSF1* siRNA or *SF3B1* siRNA and subjected to a 1 hour heat shock at 42°. Data is shown relative to mRNA levels in heat shocked cells treated with a nonsilencing control siRNA. (B) Western blot analysis of cells treated with *SF3B1* siRNA or a nonsilencing control siRNA probed with both anti-SF3B1 and anti-ACTIN antibodies. Two biologically independent replicates for each sample are shown. (C) Western blot analysis of cells treated with *SF3B1* siRNA or a nonsilencing control siRNA and probed with anti-HSP70 or anti-ACTIN antibodies. Heat shocked cells were subjected to a 1 hour at 42° and then allowed to recover for 4 hours at 37° before harvesting. Induction levels of HSP70 were quantitated by densitometry from the blots and normalized to ACTIN. Fold induction upon heat shock is shown. (D) Diagram showing the qPCR primer locations for *HSPA6* and *DNAJB1*.

Our recent genome-wide screen in *C*. *elegans* identified SF3B1 as a new positive regulator of the heat shock response (HSR) [13]. SF3B1 is a core member of the U2 snRNP and its inhibition has been shown to cause alternative splicing of a subset of transcripts in mammalian cells [22]. To explore the connections between SF3B1 and the HSR, we first tested the effect of SF3B1 depletion in cultured human cells to determine if SF3B1-mediated regulation of the HSR is conserved from worms to humans. We found that siRNA knockdown of *SF3B1* in HeLa cells led to a dramatic decrease in the cells’ ability to mount a heat shock response after exposure to standard heat shock conditions of 42° for 1 hour (Fig 1A). The HSR was measured by examining mRNA levels for both *HSPA6* and *DNAJB1*, two genes known to be regulated by HSF1 and induced by heat shock. Knockdown of *SF3B1* inhibited the HSR to 18% for *HSPA6* and 31% for *DNAJB1* relative to expression in nonsilencing siRNA control cells (Fig 1A). The extent of HSR inhibition is nearly as robust as that observed with siRNA knockdown of *HSF1*, the transcription factor that mediates the HSR. qRT-PCR analysis of the *SF3B1* transcript levels confirms knockdown of SF3B1 to greater than 95% (3.9% +/- 0.003 SEM of nonsilencing control levels) after 2 days of transfection with siRNA. Western analysis confirmed that SF3B1 protein levels dropped below the level of detection (Fig 1B). The effects of SF3B1 on the HSR were confirmed by examining the protein level of HSP70. The increase in HSP70 protein levels upon heat shock is decreased from 3.5-fold to 1.3-fold when SF3B1 levels are reduced (Fig 1C). Together, these data indicate that knockdown of SF3B1 causes a dramatic decrease in the HSR in cultured human cells. This inhibition of the HSR is unlikely to arise from a direct effect on the mRNA splicing of heat shock genes as *HSPA6* is one of the few human genes that is not spliced (Fig 1D).

### Small molecule inhibition of SF3B1 also inhibits the HSR

**Fig 2:**
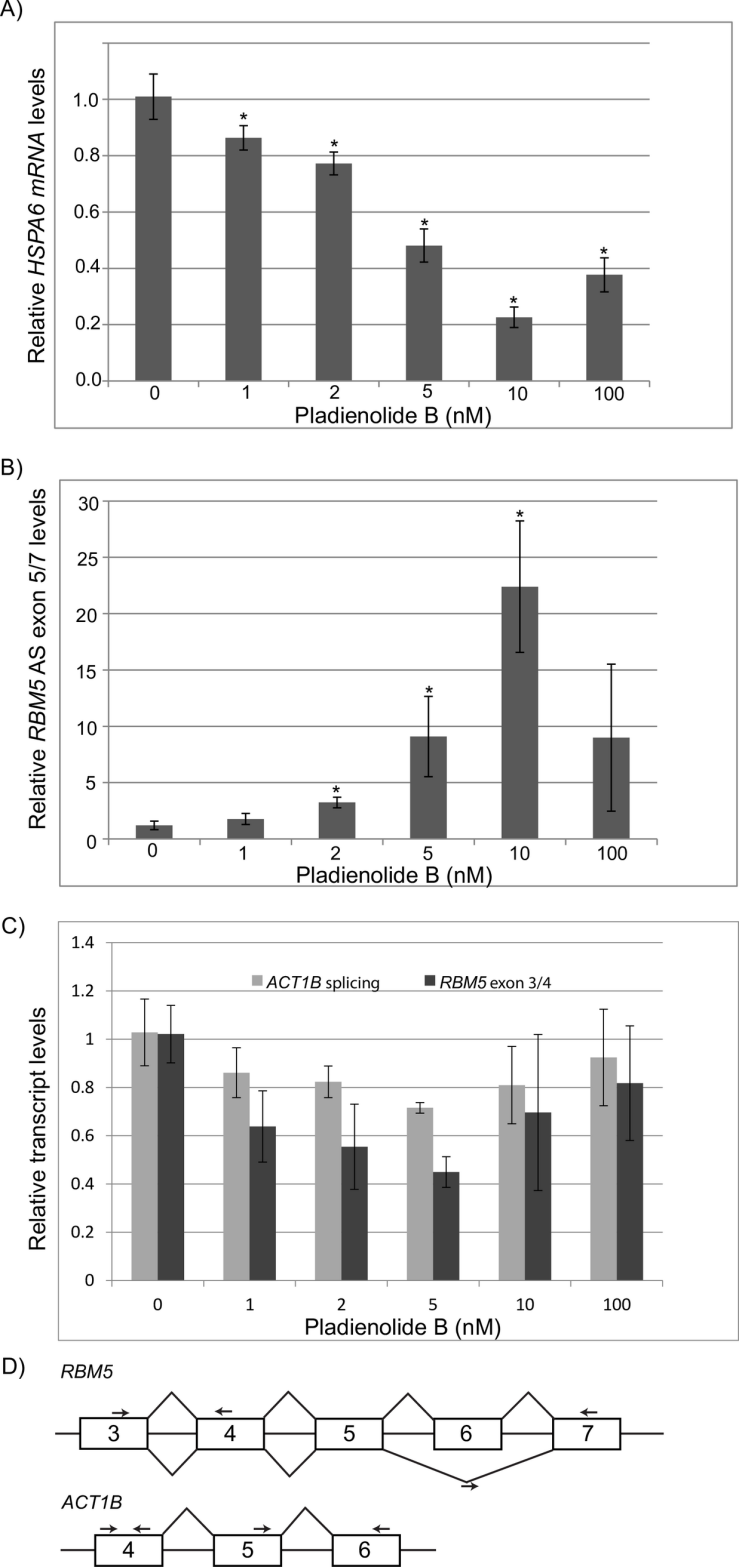
The SF3B1 inhibitor Pladienolide B inhibits the HSR in a dose-dependent manner. (A) qRT-PCR analysis of *HSPA6* mRNA levels in cells treated with varying levels of Pladienolide B (PB) and normalized to the no drug control. Cells were incubated with PB for 16 hours and then subjected to a 1 hour heat shock at 42° before harvest. (B) qRT-PCR analysis of an alternatively spliced variant of the *RBM5* transcript that is known to be sensitive to SF3B1 [22]. Cells were incubated with PB for 16 hours before harvest and normalized to the no drug control. (C) qRT-PCR analysis of the normal spliced transcript for *ACT1B* and *RBM5*. Cells were incubated with PB for 16 hours before harvest and normalized to the no drug control. (D) Diagram indicating primer locations for qPCR. * indicates p-value < 0.05.

To ensure that the effects of SF3B1 inhibition on the HSR were not an artifact of siRNA knockdown, we tested whether a small molecule inhibitor of SF3B1 could affect the HSR. A handful of naturally occurring compounds and their derivatives have been shown to directly inhibit SF3B1 activity [24]. Of these inhibitors, Pladienolide B (PB) is commercially available. Therefore, we tested the effects of exposure to PB on the HSR (Fig 2A). HeLa cells were exposed to various amounts of PB for 16 hours, heat shocked for 1 hour at 42°, then harvested for gene expression analyses via qRT-PCR (Fig 2A). A 16 hour timepoint was used as PB has been shown to cause cell cycle arrest at 24 hours and cell death after 3 days [30]. Similar to siRNA inhibition of SF3B1, inhibition of SF3B1 with PB decreases the HSR. The effects were apparent at low doses of PB, down to 1nM, which caused a modest but statistically significant 14% decrease in the HSR (p-value <0.05). A graded response was observed between 1nM and 10nM PB. At the highest concentrations of drug tested (100nM), the effect was slightly decreased, which coincided with a visibly noticeable effect on cell growth.

To validate that PB was inhibiting SF3B1, we confirmed that PB affected a previously established SF3B1 target, RBM5 [22]. Similar to the effects of PB on the HSR, a dose-response relationship was observed for PB and *RBM5* alternative splicing between 1nM and 10nM PB but a decreased effect was found at 100nM PB (Fig 2B).

These data indicate that inhibition of SF3B1 activity can be finely controlled and gives a graded but not switch-like response. The striking correlation between inhibition of the HSR and an increase in alternative splicing in response to PB suggests that the SF3B1-mediated effects on both are linked. Additionally, the response to 100nM PB indicates a fundamentally altered cellular regime in which the HSR, alternative splicing, and cell growth are all negatively affected in response to high drug conditions.

### HSF1 concentration is affected by robust but not mild levels of SF3B1 inhibition

**Fig 3:**
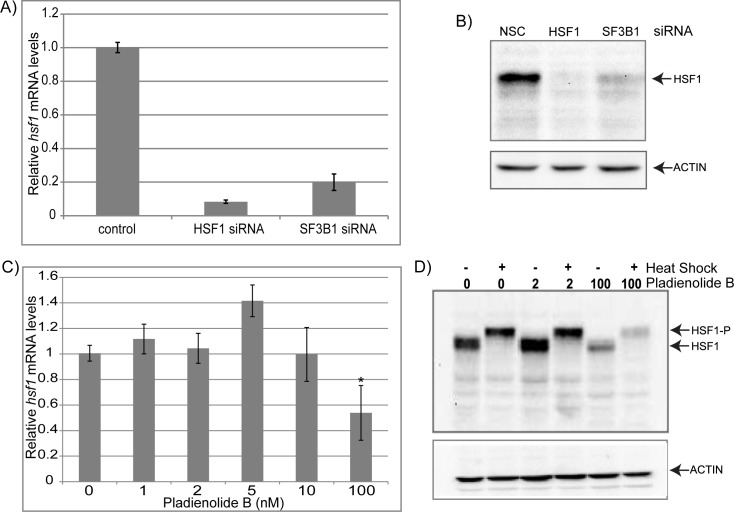
SF3B1 knockdown and high but not low levels of an SF3B1 inhibitor decreases *HSF1* mRNA and protein levels. (A) qRT-PCR analysis of *HSF1* mRNA levels in cells that have been treated with either *HSF1* siRNA or *SF3B1* siRNA relative to nonsilencing siRNA control cells. (B) Western blot analysis of cells that have been treated with non-silencing control siRNA (NSC), *HSF1* siRNA, or *SF3B1* siRNA and probed with either anti-HSF1 or anti-ACTIN antibodies. (C) qRT-PCR analysis of *HSF1* mRNA levels in cells that have been treated with varying amounts of Pladienolide B relative to control cells. (D) Western blot analysis of cells that have been treated with varying amounts of Pladienolide B, with and without heat shock at 42° for 1 hour and probed with either anti-HSF1 or anti-ACTIN antibodies.

We next investigated the mechanism by which SF3B1 regulates the HSR. The simplest hypothesis is that SF3B1, directly or indirectly, affects the expression of HSF1, the transcription factor required for the HSR. Therefore, we measured *HSF1* expression in cells knocked-down for *SF3B1* or exposed to Pladienolide B. We found that knockdown of *SF3B1* causes a significant decrease in the expression of *HSF1* mRNA (Fig 3A) and HSF1 protein (Fig 3B). Similarly, exposure to high levels of PB (100 nM) caused a decrease in expression of *HSF1* mRNA (Fig 3C) and protein (Fig 3D).Together, this data reveals that inhibition of SF3B1 blocks induction of the heat shock response by controlling the cellular levels of the HSF1 transcription factor. This effect could arise from alternative splicing of *HSF1* as 100nM PB caused a much larger decrease in HSF1 protein levels than *HSF1* mRNA levels (using primers that recognize the normally spliced transcript).

However, moderate levels of PB (1-10nM), which still cause significant decreases in the HSR, have no effect on *HSF1* mRNA (Fig 3C) or HSF1 protein levels (Fig 3D). These data demonstrate that the effects of SF3B1 on the HSR cannot be only due to regulation of HSF1 concentration, and that SF3B1 may be able to regulate the HSR by a second, distinct pathway.

### Activity of HSF1 is regulated by SF3B1

To determine if SF3B1 could affect the heat shock response through a second mechanism, we characterized the effects of SF3B1 on the different steps of the HSF1 activation pathway. The process of HSF1 activation has been well-studied: HSF1 becomes phosphorylated, translocates into the nucleus, trimerizes, and binds heat shock elements in the DNA. The final step in the pathway is activation of the HSF1 transcriptional activation domain and transcription of HSF1-dependent genes [2]. We tested the effects of SF3B1 inhibition on phosphorylation of HSF1, which is readily visible as a band shift in a western blot. We found that in both the controls and in cells exposed to Pladienolide B, all visible HSF1 protein exhibits a shift to a slower mobility upon HS, indicating that SF3B1 does not affect phosphorylation of HSF1 (Fig 3D).

**Fig 4:**
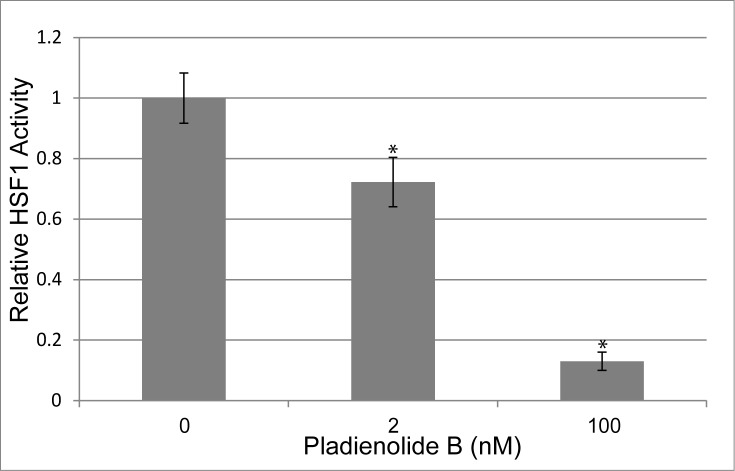
Inhibition of SF3B1 affects HSF1 transcriptional activation. Quantitation of relative luminescence from cells expressing an HSF1-Gal4 chimera containing the HSF1 activation domain and the Gal4 DNA-binding domain exposed to varying levels of Pladienolide B. Luciferase activity was monitored from a firefly luciferase reporter with a Gal4 binding element that was co-transfected with the chimera. Luciferase activity was normalized to the no drug control and a VP16-Gal4 control. Dual luciferase with a general NanoLuc reporter was also measured to correct for differences in cell density and transfection efficiency. * indicates p-value < 0.05.

To examine the final step of HSF1 activation, an established HSF1-Gal4 intronless chimera was used to isolate activation at the promoter from previous steps in the pathway [28]. This chimera fuses the activation domain of HSF1 to the Gal4 DNA binding domain, constitutively localizing it to the promoter. We tested the effects of PB in this assay by transfecting cells with the HSF1-Gal4 chimera and measuring transcriptional activity with a firefly luciferase reporter containing a Gal4 binding element. A second vector with a NanoLuc (Promega) reporter driven by the TK promoter was used as a normalization control. Since the firefly luciferase reporter has been shown to be thermosensitive and require the HSP70 heat shock protein for folding and activity, we hypothesized that inhibition of the heat shock response by PB could cause non-specific effects on the reporter system [29]. Therefore, we also normalized the effects of SF3B1 on the HSF1-Gal4 chimera to a VP16-Gal4 control to account for any nonspecific effects. After normalization, we found that cells exposed to 2nM PB displayed a statistically significant decrease in the activity of the HSF1-Gal4 construct to 67% of the no drug control (Fig 4). Incubation with 100nM PB caused a much larger decrease in HSF1 activity to 15% relative to control. These data confirm that SF3B1 does regulate the HSR through a second mechanism and that this mechanism occurs through regulation of the HSF1 transcriptional activation domain.

### The activity of SF3B1 is regulated by temperature

**Fig 5:**
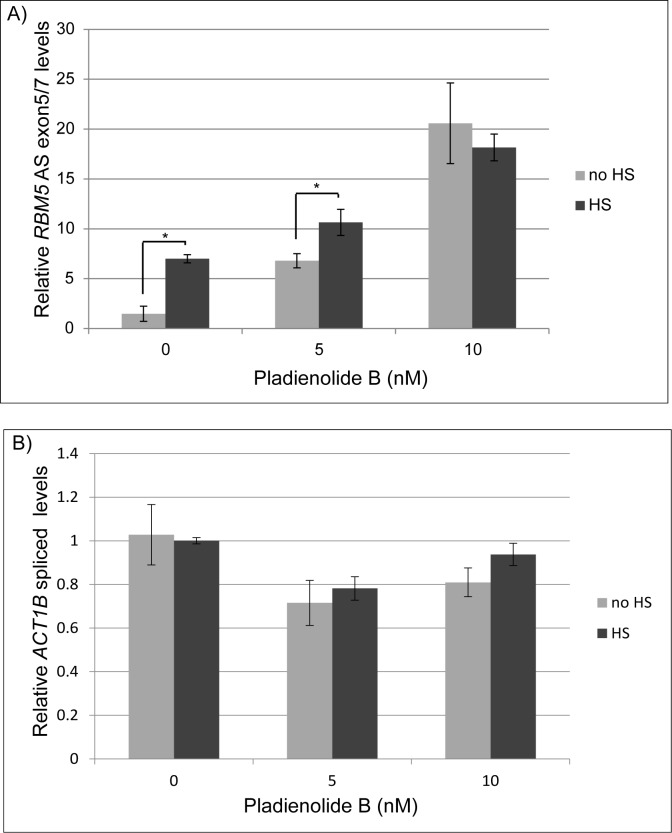
SF3B1 activity is sensitive to temperature. qRT-PCR analysis of (A) an alternatively spliced variant of the *RBM5* transcript that is known to be regulated by SF3B1 and (B) the normal splice variant of *ACT1B* in cells incubated with varying levels of PB for 16 hours +/- heat shock at 42° for 1 hour before harvest. Data is normalized to the no drug, no HS control. * indicates p-value < 0.05.

Having established that genetic and pharmacological inhibition of SF3B1 affects regulation of the HSR, we next investigated whether physiological heat shock itself could affect SF3B1 activity. Therefore, we measured the sensitivity of the established SF3B1 target, *RBM5*, upon HS. Similar to what we observed for SF3B1 knockdown and SF3B1 inhibition with PB, we found that increased temperature causes an increase in alternative splicing of *RBM5* (Fig 5A). However, increased temperature did not increase the splicing of the *ACT1B* gene (Fig 5B). The extent of alternative splicing of *RBM5* was similar to mild inhibition of SF3B1 with 5nM PB. To test whether this inhibition was dependent on SF3B1, we tested whether the increase in *RBM5* alternative splicing would still occur if SF3B1 was inhibited with PB. We found that 10nM PB eliminated the ability of temperature to further increase alternative splicing of *RBM5* (compare no HS verses HS at 10nM PB). Together, these data indicate that alternative splicing regulated by SF3B1 is a physiologically relevant response to temperature stress.

## Discussion

We have analyzed the role of SF3B1 in the regulation of the heat shock response (HSR) and found that this regulation is conserved in humans. Validating this finding, SF3B1 was also found as a regulator of the human HSR in a recent screen [31]. Moreover, we found that SF3B1 can regulate both the *concentration* of the HSF1 transcription factor as well as the *activity* of the HSF1 transcriptional activation domain. Finally, we have shown that regulation of alternative splicing by SF3B1 is a physiologically relevant response to temperature stress.

**Fig 6:**
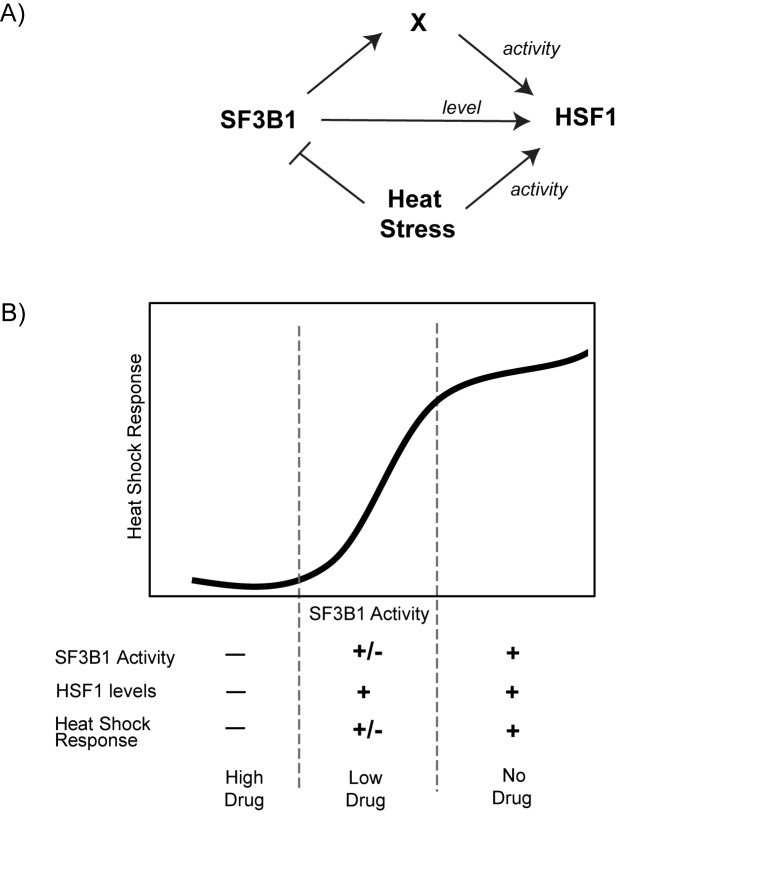
Model. (A) Model figure indicating the role of SF3B1 in regulation of the heat shock response. The central arrow indicates that SF3B1 regulates HSF1 concentration. The upper pathway indicates a second, indirect pathway where SF3B1 mediates regulation of HSF1 activity via an unknown factor X. The bottom pathway indicates heat shock stress conditions that both activate HSF1 activity and inhibit SF3B1. (B) Model figure demonstrating the relationship between the heat shock response, SF3B1 activity, and HSF1 levels.

Our data indicates that SF3B1 can both *sense* stress and can also *regulate* the stress response. Such a “sense-regulate” loop constitutes a feedback mechanism that could profoundly affect the dynamics of the heat shock response. The HSR is an extremely well-studied pathway and several distinct mathematical models that have been constructed to describe its dynamics. Our results provide a new regulatory pathway for HSR regulation that can now be incorporated into these models to improve their general utility and predictive power. The structure of the network predicted by our data forms an incoherent feedforward loop, whereby stress conditions, such as heat shock, lead to activation of the HSR in one pathway, but also inhibition of the positive HSR regulator SF3B1 via another pathway (Fig 6). Incoherent feedforward loops are common in biological systems and can function to generate a pulse of activity, which matches the transient activation that is characteristic of the heat shock response observed during mild stress conditions [32].

The importance of SF3B1-mediated HSR regulation is highlighted by our findings that this regulation occurs mechanistically at multiple levels. Regulation of HSF1 transcriptional activity is an established mechanism of HSR regulation, but the regulation of HSF1 concentration is novel. We show that both genetic inhibition of SF3B1 and chemical inhibition of SF3B1with high concentrations of Pladienolide B influence HSF1 levels, making it unlikely that this effect is a nonspecific effect from the siRNA knockdown or an off-target effect of the small molecule inhibitor. How does SF3B1 regulate both HSF1 activity and concentration? We show that the SF3B1-mediated HSR regulation is well-correlated with SF3B1-mediated alternative splicing. Therefore, the simplest model is that alternative splicing of at least two distinct SF3B1 target genes connects SF3B1 and the HSR. In agreement with this hypothesis, we show that alternative splicing regulated by SF3B1 is graded and not switch-like. As the activity of SF3B1 changes, so too does the complement of its alternatively spliced targets. We propose that the targets of SF3B1 at moderate inhibition include a factor that regulates HSF1 activity whereas strong inhibition has a distinct factor that controls HSF1 concentration.

Furthermore, a graded response in alternative splicing regulation also allows SF3B1 to be acutely sensitive to varying cellular conditions. Previous analysis of the SF3B1 regulon via splicing microarrays places a number of pathway-critical transcripts under the control of SF3B1 alternative splicing [22]. Identifying the SF3B1 regulon at different levels of activity in response to varying environmental conditions should help illuminate how SF3B1 and the spliceosome co-ordinate a unified cellular response to changing conditions.

It has been known for decades that the spliceosome is sensitive to heat shock. By showing that splicing is coordinated with the HSR, our findings introduce the intriguing possibility that other stress-sensitive processes might also be linked to the HSR. Moreover, splicing is sensitive to other stress conditions, opening up the possibility that alternative splicing by SF3B1 could be sensitive to other stresses and/or regulate other protective stress response pathways. Taken together, coordination between splicing and the HSR might be the tip of the iceberg in an integrated network of coordinated stress-sensitive processes and cytoprotective stress responses.

Our finding that SF3B1 can regulate HSF1 concentration could have profound implications for cancer biology. For example, high levels of HSF1 are found in cancer and correlated with negative outcomes [33,34]. Unfortunately, little attention has been given to regulation of HSF1 concentration as the levels of HSF1 do not significantly change during acute HS stress. How does the cell establish the typically constant level of HSF1 concentration and how is this regulation subverted during cancer? We have shown here that robust inhibition of SF3B1 leads to a decrease in HSF1 levels. Aberrant forms of SF3B1 are also commonly associated with cancer and are predicted to be gain-of-function [35–37]. Therefore, we propose that SF3B1 mutations could be driving increased HSF1 levels in some cancers. How SF3B1 and HSF1 influence each other in the highly mutated landscape of a transformed cell and how the relationship between SF3B1 and HSF1 are affected in different malignancies could provide valuable clues to basic systems biology and to cancer biology.

## Supporting information

S1 Table Data supporting each figure displayed in the manuscript. (XLSX)
